# Effects of Different Dietary Protein Levels on the Growth Performance, Serum Biochemical Parameters, Fecal Nitrogen, and Carcass Traits of Huanjiang Mini-Pigs

**DOI:** 10.3389/fvets.2021.777671

**Published:** 2021-12-20

**Authors:** Xichen Zhao, Yating Liu, Hao Ding, Pan Huang, Yulong Yin, Jinping Deng, Xiangfeng Kong

**Affiliations:** ^1^Guangdong Laboratory of Lingnan Modern Agriculture, Guangdong Provincial Key Laboratory of Animal Nutrition Control, and National Engineering Research Center for Breeding Swine Industry, College of Animal Science, South China Agricultural University, Guangzhou, China; ^2^Hunan Provincial Key Laboratory of Animal Nutrition Physiology and Metabolism Process, Institute of Subtropical Agriculture, Chinese Academy of Sciences, Changsha, China; ^3^National Center of Technology Innovation for Synthetic Biology, Tianjin Institute of Industrial Biotechnology, Chinese Academy of Sciences, Tianjin, China; ^4^Research Center of Mini-Pig, Huanjiang Observation and Research Station for Karst Ecosystems, Chinese Academy of Sciences, Huanjiang, China

**Keywords:** carcass traits, dietary protein, fecal nitrogen content, growth performance, Huanjiang mini-pig, serum biochemical parameters

## Abstract

The Huanjiang mini-pig is a Chinese local breed and, the optimal dietary crude protein (CP) level for this breed is still unknown. Therefore, the present study was conducted to investigate its optimum dietary CP level upon the growth performance, serum biochemical parameters, fecal nitrogen content, and carcass traits. Three independent trials with 360 pigs were included. A total of 220 pigs (5.32 ± 0.46 kg) were fed *ad libitum*, either a 14, 16, 18, 20, or 22% CP diet from about 5- to 10-kg (trial 1); 84 pigs (11.27 ± 1.43 kg) were fed either a 12, 14, 16, 18, or 20% CP diet from about 10- to 20-kg (trial 2); and 56 pigs (18.80 ± 2.21 kg) were fed either a 10, 12, 14, 16, or 18% CP diet from about 20- to 30-kg (trial 3). In trial 1, as dietary CP levels increased, the feed-to-gain ratio (F/G) quadratically decreased (*p* < 0.05) and was minimal at the 18.42% CP level. The average daily feed intake (ADFI) and final body weight (BW) were not affected by dietary CP levels while the fat percentage decreased (*p* < 0.05). Besides, a linear decrease in slaughter rate (*p* = 0.06) and a linear increase in bone percentage (*p* < 0.05), serum urea nitrogen (UN) (*P* < 0.05), and fecal nitrogen content (*p* = 0.07) of pigs were observed. In trial 2, as dietary CP levels increased, the average daily gain quadratically increased (*p* < 0.05) and was maximum at the 16.70% CP level. The slaughter rate linearly decreased (*p* < 0.05) whereas the skin rate, serum UN, and NH_3_-N increased (*p* < 0.05) linearly, as well as fecal nitrogen content (*p* = 0.06). In trial 3, as dietary CP levels increased, the F/G increased (*p* < 0.05), while the ADFI and ADG quadratically decreased (*p* < 0.05) and was maximum at nearly 14.00% CP level. The bone percentage and serum UN increased (*p* < 0.05) linearly but the slaughter rate decreased (*P* < 0.05) linearly, and the fecal nitrogen content quadratically decreased (*p* = 0.07) whereas the albumin increased (*p* < 0.05) quadratically. Taken together, the optimal dietary CP levels for growth performance of Huanjiang mini-pigs from 5- to 10-kg, 10- to 20-kg, and 20- to 30-kg were 18.42, 16.70, and 14.00%, respectively.

## Introduction

During the past decades, a large number of western commercial pig breeds, including Duroc and Yorkshire have been imported into China. These breeds exhibited higher feed efficiency and growth rate than did Chinese domestic pig breeds, contributing nearly 98% of the market in China (1). According to the data publicized by the Ministry of Agriculture and Rural Affairs of China, nearly 25 Chinese local breeds of pig are disappearing and eight breeds have died. With the spread of the African swine fever into China in 2018 (2), the Chinese indigenous pig breeds have faced a more serious situation. Predictably, accelerating the industrial utilization of Chinese local pig breeds could be one of the crucial strategies for their preservation.

The Huanjiang mini-pig, a famous local pig breed originated from Huanjiang county, Guangxi province, China, has similar physiology profiles to human (3). In addition, this mini-pig was famous for its superior meat quality. Our previous studies mainly focused on nutritional regulation on the intestinal health of Huanjiang mini-pigs during the pregnancy (4–7), suckling (3, 8, 9), and weaning periods (10–12). However, few studies have focused on the growth and development of weaned or nursery pigs.

It is well-known that the high growth rate of weaned piglets is maintained (13), and their growth rate has a profound impact on the subsequent growing (14). Dietary protein, as one of the major macronutrients, is critical for the growth, development, and health status of pigs (15, 16). However, a higher or lower dietary crude protein (CP) level might cause several adverse outcomes, such as bowel disease (17). Therefore, the optimal dietary CP level is essential to support the growth of pigs and then increase the economic benefits of large-scale farming.

The dietary CP level used by local pig farms might not meet the requirement for Huanjiang mini-pigs due to the fact that it was lower than the recommended value of nutrient requirements of swine (18) and the Chinese nutrient requirements of swine in China (NY/T65-2004) [(19); Supplementary Figure S1]. Therefore, the present study was conducted to investigate the effects of dietary CP levels on growth performance, carcass traits, serum biochemical parameters, and fecal nitrogen content of Huanjiang mini-pigs during different growth stages, and then the optimal dietary CP levels for the Huanjiang mini-pigs would be evaluated.

## Materials and Methods

### Experimental Animals, Treatments, and Housing

The present study was conducted in Bamian village (E108.41265°, N 25.09043°), Huanjiang county, Guangxi province, China. A completely random block single-factor design was carried out, and a total of 360 Huanjiang mini-pigs (half barrows and half gilts) were selected. This study included three independent trials which were distinguished by the growth stages of Huanjiang mini-pigs. During the 5- to 10-kg growth stage (trial 1), a total of 220 Huanjiang mini-pigs (5.32 ± 0.46 kg) were chosen according to their body weight (BW) and gender and then divided into five dietary groups with 14, 16, 18, 20, or 22% CP, and each diet group had 8–10 replicates (pens) with five pigs per pen. During the 10- to 20-kg growth stage (trial 2), 84 Huanjiang mini-pigs (11.27 ± 1.43 kg) were chosen and divided into five dietary groups with 12, 14, 16, 18, or 20% CP, and each group had 15–19 replicates (pens) with one pig per pen. During the 20- to 30-kg growth stage (trial 3), 56 Huanjiang mini-pigs (18.80 ± 2.21 kg) were used and divided into five dietary groups with 10, 12, 14, 16, or 18% CP, and each group had 11–12 replicates (pens) with one pig per pen.

All the trials had a 5-day adaptation period, and the period of three trials lasted 28, 28, and 26 days, respectively. All the pigs were fed three times per day (08:00, 14:00, and 20:30) and had *ad libitum* access to feed and water. The pigs were fed with the diets used by the local pig farm during the adaptation period and fed with the corresponding experimental diets during the trial period. The experimental diets (corn-soybean based meal) were formulated using the recommended values of individual ingredients to be isoenergetic according to the National Research Council (18), as shown in Tables 1–3. The dietary CP levels were designed referring to both the nutrient requirements of swine (18) and the Chinese nutrient requirements of swine (19). The temperature and relative humidity in the feeding house were 26.0 ± 3.4°C and 74.3 ± 11.2%, respectively.

**Table 1:** Ingredients and nutrient levels of the experimental diets for the 5- to 10-kg Huanjiang-mini pigs (air-dried).

**Items**	**CP levels, %**				
	**14**	**16**	**18**	**20**	**22**
**Ingredients, %**					
Corn	61.20	56.20	51.80	47.50	42.70
Soybean meal (43.9%)	5.70	10.10	13.20	15.60	18.80
Fish meal	2.00	2.00	2.00	2.00	2.00
Wheat bran	9.00	9.00	9.00	9.00	9.00
Whey powder (85% lactose)	10.00	10.00	10.00	10.00	10.00
Extruded full-fat soybean	5.00	5.00	5.00	5.00	5.00
Soy protein concentrate	1.00	2.10	4.00	6.30	8.20
Corn oil	0.40	0.20			
*L*-Lys (98.5%)	1.10	0.90	0.75	0.55	0.40
*DL*-Met (99%)	0.20	0.15	0.15	0.10	0.10
*L*-Thr (99%)	0.40	0.35	0.25	0.20	0.10
*L*-Trp (99%)	0.10	0.10	0.05	0.05	
Dicalcium phosphate	1.40	1.40	1.30	1.20	1.20
Premix^a^	2.50	2.50	2.50	2.50	2.50
Total	100.00	100.00	100.00	100.00	100.00
**Nutrient levels** ^**b**^					
ME, MJ/kg	13.73	13.69	13.68	13.72	13.74
CP	14.09	16.10	18.10	20.07	22.08
SID Lys	1.36	1.34	1.36	1.34	1.36
SID Met	0.39	0.37	0.39	0.37	0.40
SID Thr	0.76	0.79	0.77	0.80	0.77
SID Trp	0.21	0.24	0.22	0.24	0.22
Ca	0.79	0.80	0.80	0.79	0.80
TP	0.66	0.69	0.69	0.70	0.72

a *Provided the following per kg of the diet: Cu, 128 mg; Mn, 97.6 mg; Zn 109 mg; Fe, 197.6 mg; Se, 1 mg; I, 1 mg; Co, 1 mg; VA, 32,500 IU; VD_3_, 10,000 IU; VE, 80 IU; VK_3_, 10 mg; VB_1_, 10 mg/kg; VB_2_, 25 mg; VB_6_, 8 mg; VB_12_, 0.075 mg; biotin, 0.075 mg; folic acid, 5 mg; nicotinamide, 100 mg; pantothenic acid, 50 mg; choline, 1,600 mg; limestone, 0.80%; NaCl, 0.30%; mildewcide, 0.10%; ethoxyquinoline (33%), 0.05%; acidifier, 0.25%*.

b *Calculated according to the nutrient requirements of swine (NRC, 2012); CP, crude protein; Lys, Lysine; ME, metabolic energy; Met, Methionine; SID, standard ileal digestible; Thr, Threoine; TP, total phosphorus; Trp, Tryptophan*.

**Table 2:** Ingredients and nutrient levels of the experimental diets for the 10- to 20-kg Huanjiang-mini pigs (air-dried).

**Items**	**CP levels, %**				
	**12**	**14**	**16**	**18**	**20**
**Ingredients, %**					
Corn	72.10	66.50	60.90	55.45	49.80
Soybean meal (43.9%)	0.50	6.75	12.95	19.00	25.25
Soybean meal (fermented)	4.00	4.00	4.00	4.00	4.00
Fish meal	2.00	2.00	2.00	2.00	2.00
Wheat bran	8.00	8.00	8.00	8.00	8.00
Whey powder (85% lactose)	5.00	5.00	5.00	5.00	5.00
Soybean oil	0.80	0.60	0.40	0.20	
*L*-Lys (98.5%)	1.10	0.95	0.75	0.60	0.40
*DL*-Met (99%)	0.20	0.15	0.15	0.10	0.10
*L*-Thr (99%)	0.45	0.35	0.30	0.20	0.10
*L*-Trp (99%)	0.15	0.10	0.05	0.05	
Dicalcium phosphate	1.20	1.10	1.00	0.90	0.85
Premix^a^	4.50	4.50	4.50	4.50	4.50
Total	100.00	100.00	100.00	100.00	100.00
**Nutrient levels** ^**b**^					
ME, MJ/kg	13.73	13.68	13.64	13.59	13.54
CP	12.06	14.08	16.10	18.08	20.08
SID Lys	1.22	1.24	1.22	1.24	1.23
SID Met	0.38	0.35	0.38	0.35	0.38
SID Thr	0.74	0.72	0.75	0.73	0.71
SID Trp	0.23	0.21	0.19	0.22	0.20
Ca	0.70	0.70	0.70	0.69	0.70
TP	0.59	0.60	0.61	0.61	0.63

a *Provided the following per kg of the diet: Cu, 128 mg; Mn, 97.6 mg; Zn, 109 mg; Fe, 197.6 mg; Se, 1 mg; I, 1 mg; Co, 1 mg; VA, 32,500 IU; VD_3_, 10,000 IU; VE, 80 IU; VK_3_, 10 mg; VB_1_, 10 mg/kg; VB_2_, 25 mg; VB_6_, 8 mg; VB_12_, 0.075 mg; biotin, 0.075 mg; folic acid, 5 mg; nicotinamide, 100 mg; pantothenic acid, 50 mg; choline, 1,600 mg; limestone, 0.80%; sucrose, 2%; NaCl, 0.3%; mildewcide, 0.10%; ethoxyquinoline (33%), 0.05%; acidifier, 0.25%*.

b *Calculated according to the nutrient requirements of swine (NRC, 2012); CP, crude protein; Lys, Lysine; ME, metabolic energy; Met, Methionine; SID, standard ileal digestible; Thr, Threoine; TP, total phosphorus; Trp, Tryptophan*.

**Table 3:** Ingredients and nutrient levels of the experimental diets for the 20- to 30-kg Huanjiang-mini pigs (air-dried).

**Items**	**CP levels, %**				
	**10**	**12**	**14**	**16**	**18**
**Ingredients, %**					
Corn	69.65	64.25	58.65	53.35	47.60
Soybean meal (43.9%)	1.00	6.90	13.20	19.10	25.50
Soybean meal (fermented)	2.00	2.00	2.00	2.00	2.00
Wheat bran	12.00	12.00	12.00	12.00	12.00
Rice hull and bran^a^	5.00	5.00	5.00	5.00	5.00
Soybean oil	3.00	2.80	2.60	2.30	2.10
*L*-Lys (98.5%)	0.95	0.80	0.60	0.45	0.25
*DL*-Met (99%)	0.15	0.15	0.10	0.10	0.05
*L*-Thr (99%)	0.35	0.30	0.20	0.15	0.05
*L*-Trp (99%)	0.10	0.10	0.05	0.05	
Dicalcium phosphate	1.10	1.00	0.90	0.80	0.75
Premix^b^	4.70	4.70	4.70	4.70	4.70
Total	100.00	100.00	100.00	100.00	100.00
**Nutrient levels** ^**c**^					
ME, MJ/kg	13.27	13.23	13.18	13.12	13.06
CP	10.12	12.11	14.10	16.10	18.13
SID Lys	1.00	1.01	1.00	1.01	1.00
SID Met	0.29	0.31	0.29	0.31	0.29
SID Thr	0.59	0.61	0.59	0.62	0.60
SID Trp	0.17	0.20	0.18	0.21	0.19
Ca	0.67	0.67	0.66	0.66	0.67
TP	0.54	0.54	0.55	0.56	0.57

a *Nutritional levels were referred to the China Feed Database Information Network Centre (the 29th edition)*.

b *Provided the following per kg of the diet: Cu, 128 mg; Mn, 97.6 mg; Zn, 109 mg; Fe, 197.6 mg; Se, 1 mg; I, 1 mg; Co, 1 mg; VA, 32,500 IU; VD_3_, 10,000 IU; VE, 80 IU; VK_3_, 10 mg; VB_1_, 10 mg/kg; VB_2_, 25 mg; VB_6_, 8 mg; VB_12_, 0.075 mg; biotin, 0.075 mg; folic acid, 5 mg; nicotinamide, 100 mg; pantothenic acid, 50 mg; choline, 1,600 mg; limestone, 1%; sucrose, 2%; NaCl, 0.3%; mildewcide, 0.10%; ethoxyquinoline (33%), 0.05%; acidifier, 0.25%*.

c *Calculated according to the nutrient requirements of swine (NRC, 2012); CP, crude protein; ME, metabolic energy; SID, standard ileal digestible; TP, total phosphorus*.

### Measurement of Growth Performance

The daily feed intake of pigs was recorded at 07:30 per pen. On the first day of the 5-day adaptation period, the individual pig was weighted for the initial BW; at the end of the trial period, the pigs were weighted for the final BW. All the pigs were weighted after a 12-h fasting. The initial and final BW were used to calculate the average daily gain (ADG), while the feed-to-gain ratio (F/G) was calculated based on the daily feed intake and ADG.

### Sampling and Chemical Analyses

At the end of each trial, blood samples were collected from the jugular veins using 10-ml centrifuge tubes and centrifuged at 3,000 × *g* and 4°C for 15 min to recover the serum. Then, the samples were stored at −20°C until biochemical analyses. The contents of total protein (TP), albumin, urea nitrogen (UN), and NH_3_-N in serum were measured using a Cobas C311 automatic biochemical analyzer (Roche, Basel, Switzerland) and respective commercial assay kits (Leadman, Beijing, China).

During the 5 days before each trial ended, nearly 100 g of fresh feces was collected per pen per day and then added into 10% H_2_SO_4_ solution (W/V, 1:10). The feces collected were well-mixed according to the pens, dried in a forced air oven at 60°C for 72 h, and then ground through a 1-mm screen. The fecal samples were stored at −20°C before further analyses (20). The fecal nitrogen content was determined using a Mobile Injection Analyzer (AA3, Seal, Norderstedt, Germany).

### Assessment of Carcass Traits

At the end of each trial, one pig per pen was slaughtered quickly by severance of the jugular veins after an electric shock (120 V, 200 Hz), and then carcass traits were determined according to the ZZWH01-200 standard (China). The leaf fat was removed, carcasses were split down the midline, and hot carcass weights were recorded. The carcass' straight length and slanting length were measured with a tape line. The backfat depth (the first rib, the 6th and 7th ribs, and last lumbar vertebra) and eye muscle area (the 6th and 7th ribs) were measured using a vernier caliper. The average backfat thickness was calculated from the individual measurement. The slaughter rate was calculated according to the collected weights referred to by Zou et al. (21). The eye muscle area was calculated by area = (height × width) × 0.7. The muscle, fat, bone, and skin of the pigs were stripped and weighted to calculate their percentages of weight to carcass weight.

### Statistical Analyses

The pen was used as the basic experimental unit for statistical analysis. Statistical analyses for the growth performance, carcass traits, serum biochemical parameters, and fecal nitrogen content were performed using the one-way ANOVA procedure of SAS software (SAS 8.1, Institute, Inc., Cary, NC, USA), and a significant difference was obtained by Duncan's multiple comparisons. When data did not comply with the normal distribution or homogeneity, the significance was conducted by the Kruskal–Wallis test. The linear and quadratic regression analyses were performed using the GraphPad Prism 7.0 (GraphPad Software, San Diego, CA, USA) and PROC REG of SAS software, respectively. Results are presented as means ± pooled standard error of the mean (SEM). Statistical probability values <0.05 were considered significant, and 0.05 ≤ *p* < 0.10 was considered a trend of difference.

## Results

### Effects of Dietary CP Levels on Growth Performance of Huanjiang Mini-Pigs During Different Growth Stages

**Figure 1:**
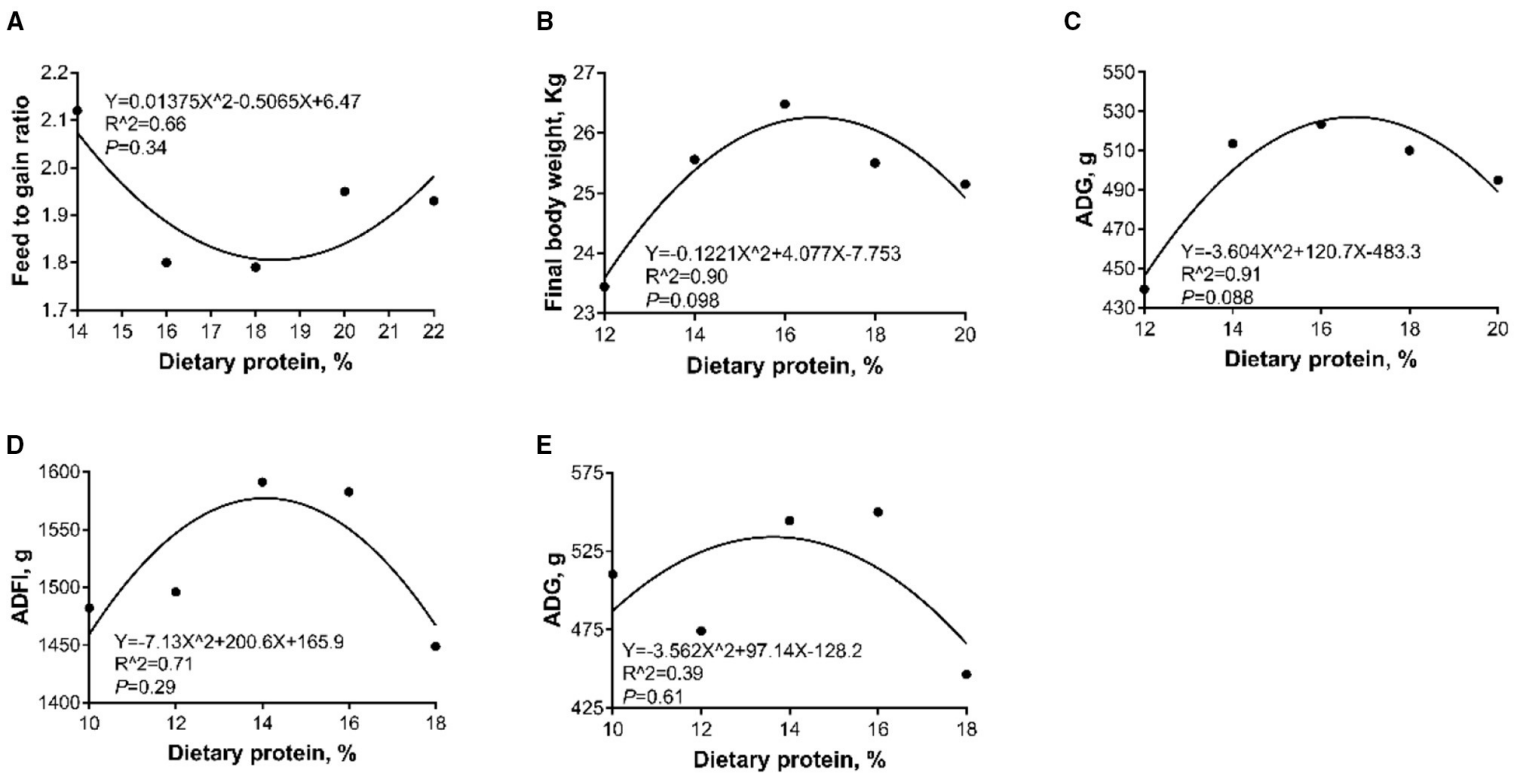
Quadratic regression analysis for the relations between the dietary crude protein (CP) levels and feed-to-gain ratio (F/G) (**A**, 5- to 10-kg), final body weight (**B**, 10- to 20-kg), average daily gain (ADG) (**C**, 10- to 20-kg), average daily feed intake (ADFI) (**D**, 20- to 30-kg), and ADG (**E**, 20- to 30-kg) of Huanjiang mini-pigs. The minimal point for **(A)** is (18.42, 1.806); the maximum point for **(B)** is (16.70, 26.28), **(C)** is (16.75, 527.28), **(D)** is (14.07, 1576.85), and **(E)** is (13.64, 534.08).

As shown in Table 4, during the 5- to 10-kg growth stage, no significant difference was observed in the final BW, ADFI, and ADG among the five groups (*p* > 0.05). The F/G in the 16 and 18% CP diet groups was lower (*p* < 0.05) than that in the 14% CP diet group. A notably quadratic relation was observed between the dietary CP levels and F/G (*p* < 0.05). The minimal F/G (1.806) was achieved at 18.42% CP level (Figure 1A).

**Table 4:** Effects of dietary crude protein (CP) levels on the growth performance of Huanjiang mini-pigs from 5- to 30-kg^1^.

**Items^2^**	**CP levels, %**					**SEM**	***p-*** **values^3^**		
							**D**	**L**	**Q**
**5- to 10-kg stage**	14	16	18	20	22				
Initial BW, kg	5.39	5.33	5.29	5.27	5.31	0.07	0.99	0.65	0.85
Final BW, kg	13.12	13.31	13.73	12.19	14.14	0.43	0.68	0.87	0.92
ADFI, g	516.51	566.28	556.11	495.2	589.72	24.27	0.63	0.77	0.94
ADG, g	273.12	294.47	320.76	273.63	312.1	11.94	0.63	0.52	0.75
F/G	2.12^A^	1.80^B^	1.79^B^	1.95^AB^	1.93^AB^	0.04	0.049	0.50	0.04
**10- to 20-kg stage**	12	14	16	18	20				
Initial BW, kg	11.32	11.21	11.21	11.29	11.33	0.16	1.00	0.93	0.96
Final BW, kg	23.44	25.56	26.48	25.5	25.15	0.40	0.13	0.16	0.04
ADFI, g	1292.65	1374.88	1420.97	1469.29	1375.43	23.24	0.14	0.08	0.04
ADG, g	439.55^b^	513.61^a^	523.57^a^	510.15^a^	495^ab^	11.01	0.07	0.10	0.02
F/G	2.96	2.68	2.81	2.89	2.80	0.05	0.52	0.65	0.67
**20- to 30-kg stage**	10	12	14	16	18				
Initial BW, kg	18.76	18.85	18.78	18.83	18.79	0.30	1.00	0.98	1.00
Final BW, kg	32.03^ab^	31.19^b^	32.01^ab^	33.59^a^	30.50^b^	0.42	0.08	0.92	0.34
ADFI, g	1482.26^AB^	1496.04^AB^	1591.23^A^	1582.83^A^	1448.76^B^	17.93	0.03	0.84	0.03
ADG, g	510.31^AB^	474.31^BC^	509.02^AB^	549.88^A^	450.35^C^	9.01	<0.01	0.42	0.02
F/G	2.93^B^	3.18^AB^	2.96^AB^	2.89^B^	3.22^A^	0.04	0.045	0.33	0.47

1 *Different capital letters indicate differed significantly among the treatments (p < 0.05), and different lowercase indicate a trend of difference (0.05 ≤ P < 0.10); n = 6–8 (5- to 10-kg), n = 7–9 (10- to 20-kg), n = 10–11 (20- to 30-kg)*.

2 *ADFI, average daily feed intake; ADG, average daily gain; BW, body weight; F/G, feed-to-gain ratio*.

3 *D, diet; L, linear; Q, quadratic*.

During the 10- to 20-kg growth stage, although significant differences in final BW and ADFI were not observed, the 12% CP diet group displayed a lower trend (*p* = 0.07) in ADG than that of the 14, 16, and 18% CP diet groups. The final BW (*R*^2^ = 0.90, *p* = 0.098) and ADG (*R*^2^ = 0.91, *p* = 0.088) quadratically increased as the dietary CP levels increased (*p* < 0.05). The optimal final BW and ADG were obtained when the dietary CP levels were 16.70 and 16.75%, respectively (Figures 1B,C). No significant difference was observed in F/G among treatment groups (*p* > 0.05).

During the 20- to 30-kg growth stage, the 14 and 16% CP diet groups had a higher ADFI than the 18% CP diet group (*p* < 0.05). As a result, a higher ADG was observed in the 14 and 16% CP diet groups (*p* < 0.05) and thus contributed a higher final BW (*p* = 0.08). A notably quadratic relationship between dietary CP levels and ADFI and ADG was found (*p* < 0.05), and the highest values of ADFI and ADG were achieved when the dietary CP levels were 14.07 and 13.64%, respectively (Figures 1D,E). Besides, the F/G in the 18% CP diet group was higher (*p* < 0.05) than that of the 10 and 16% CP diet groups.

### Effects of Dietary CP Levels on Serum Biochemical Parameters and Fecal Nitrogen Content of Huanjiang Mini-Pigs During Different Growth Stage

As shown in Table 5, during the 5- to 10-kg growth stage, the levels of serum UN in the 20 and 22% CP diet groups were higher than those of other diet groups (*p* < 0.05). The serum content of TP of the 22% CP diet group had a tendency to be lower than that of the 14 and 20% CP diet groups (*p* = 0.06). During the 10- to 20-kg growth stage, the serum contents of UN and NH_3_-N in the 20% CP diet group were higher than those of other diet groups (*p* < 0.05). Furthermore, the contents of serum UN in the 16 and 18% CP diet groups were higher than those of 12 and 14% CP diet groups (*p* < 0.05). During the 20- to 30-kg growth stage, the content of serum albumin in the 10% CP diet group was lower than that of the other groups (*p* < 0.05). Besides, the contents of serum UN in the 10 and 12% CP diet groups were lower than those of other diet groups (*p* < 0.05).

**Table 5:** Effects of dietary crude protein (CP) levels on the serum biochemical parameters of Huanjiang mini-pigs from 5- to 30-kg^1^.

**Items^2^**	**CP levels, %**					**SEM**	***p*** **-values^3^**		
							**D**	**L**	**Q**
**5- to 10-kg stage**	14	16	18	20	22				
Total protein, g/L	68.99^a^	65.28^ab^	64.51^ab^	68.26^a^	63.16^b^	0.75	0.06	0.14	0.31
Albumin, g/L	37.90	39.88	43.12	40.56	39.57	0.73	0.23	0.44	0.10
UN, mmol/L	2.89^B^	2.03^B^	2.86^B^	4.44^A^	4.62^A^	0.23	<0.01	<0.01	<0.01
NH_3_-N, μmol/L	242.49	227.91	165.88	169.68	166.67	13.04	0.28	0.02	0.04
**10- to 20-kg stage**	12	14	16	18	20				
Total protein, g/L	68.71	69.09	69.80	70.47	68.22	0.65	0.86	0.90	0.63
Albumin, g/L	45.60	46.79	47.91	47.74	44.90	0.69	0.54	0.93	0.29
UN, mmol/L	3.20^C^	3.59^C^	4.73^B^	5.75^B^	7.64^A^	0.30	<0.01	<0.01	<0.01
NH_3_-N, μmol/L	169.78^B^	186.60^B^	172.82^B^	180.61^B^	414.80^A^	20.10	0.03	<0.01	<0.01
**20- to 30-kg stage**	10	12	14	16	18				
Total protein, g/L	66.61	74.55	72.03	72.14	72.39	1.02	0.17	0.25	0.17
Albumin, g/L	43.83^B^	51.35^A^	48.55^A^	49.94^A^	47.59^AB^	0.77	0.01	0.31	0.02
UN, mmol/L	3.47^B^	3.74^B^	4.70^A^	5.18^A^	5.10^A^	0.18	<0.01	0.03	0.08
NH_3_-N, μmol/L	222.90	330.30	380.20	411.34	437.23	30.42	0.16	<0.01	<0.01

1 *Different capital letters indicate differ significantly among the treatments (p < 0.05), and different lowercase indicate a trend of difference (0.05 ≤ P < 0.10); n = 6–9 (5- to 10-kg), n = 7–9 (10- to 20-kg), n = 6–8 (20- to 30-kg)*.

2 *UN, urea nitrogen*.

3 *D, diet; L, linear; Q, quadratic*.

**Figure 2:**
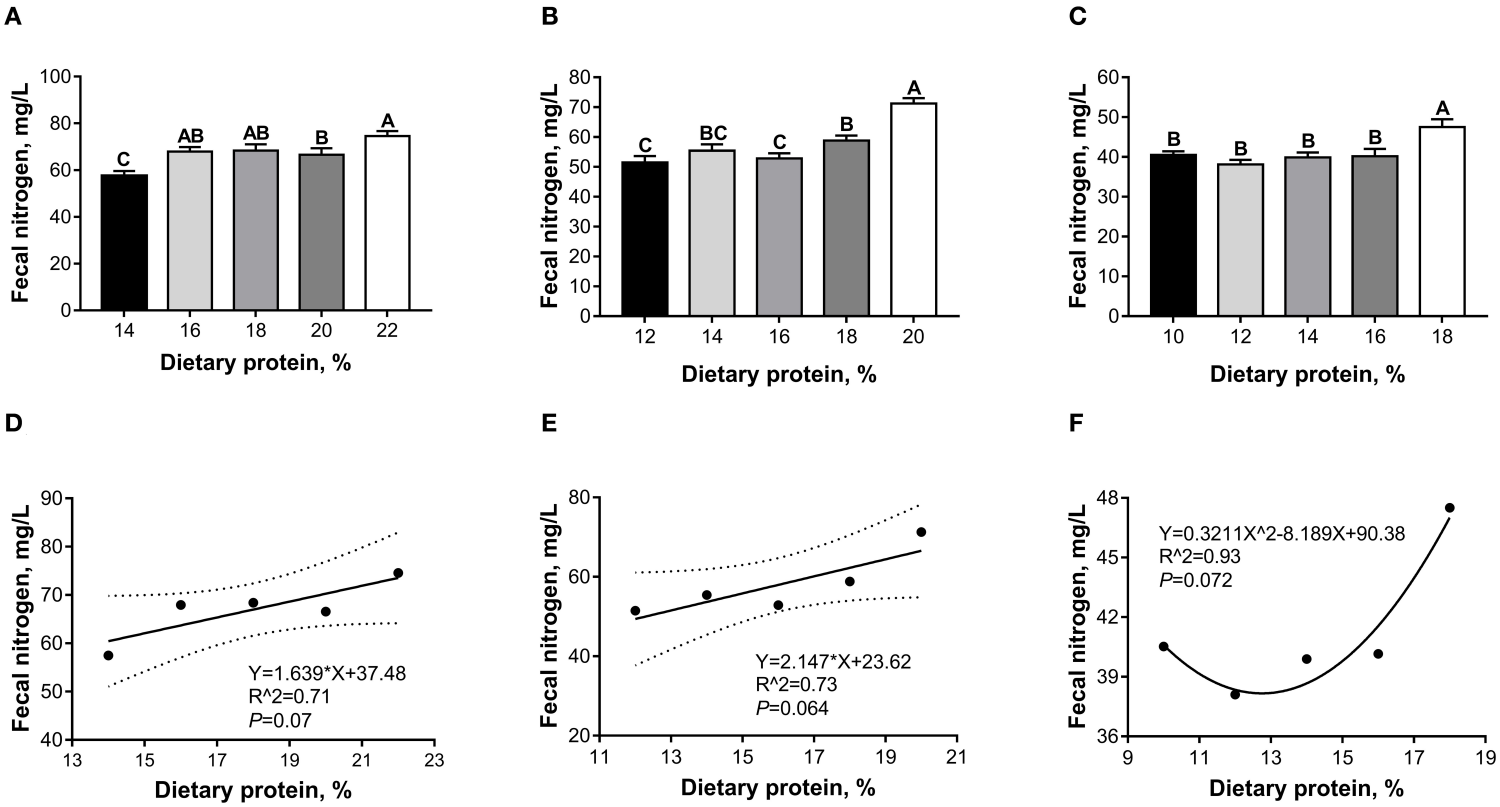
Effects of dietary crude protein (CP) levels on total nitrogen content in feces of Huanjiang mini-pigs in 5- to 10-kg (**A**, *n* = 6–8), 10- to 20-kg (**B**, *n* = 7–8), and 20- to 30-kg (**C**, *n* = 7–9) growth stage. Regression analysis for the relation between dietary CP levels and fecal nitrogen content of Huanjiang mini-pigs in the 5- to 10-kg **(D)**, 10- to 20-kg **(E)**, and 20- to 30-kg **(F)** growth stages. Different uppercase lettered on the bar indicates significant difference. The dotted lines in **(D,E)** represent the confidence interval of 95%, and the minimal point for **(F)** is (12.75, 38.17).

As shown in Figure 2, during the 5- to 10-kg growth stage, the fecal nitrogen content in the 14% CP diet group was lower (*p* < 0.05) than those of other groups (Figure 2A). During the 10- to 20-kg growth stage, the 20% CP diet group had a higher (*p* < 0.05) fecal nitrogen content than the other groups, and the 18% CP diet group had a higher (*p* < 0.05) fecal nitrogen content than the 12 and 16% CP diet groups (Figure 2B). During the 20- to 30-kg growth stage, the fecal nitrogen content in the 18% CP diet group was higher (*p* < 0.05) than the other groups (Figure 2C).

### Effects of Dietary CP Levels on Carcass Traits of Huanjiang Mini-Pigs During Different Growth Stages

As shown in Table 6, during the 5- to 10-kg growth stage, the 14% CP diet group tended to increase the skin weight (*p* = 0.07) and skin rate (*p* = 0.06) compared with the 18 and 20% CP diet groups. A notably quadratic relation between the dietary CP levels and skin weight and skin rate was found (*p* < 0.05). The lean meat percentage and bone percentage were increased (*p* < 0.05) as the dietary CP levels increased. The lean meat percentage of the 18% CP diet group was higher (*p* < 0.05) than the 14 and 16% CP diet groups, and the bone percentage of the 18, 20, and 22% CP diet groups was higher (*p* < 0.05) than the 14% CP group. Conversely, the fat percentage was decreased linearly, and the 18, 20, and 22% CP diet groups was lower (*p* < 0.05) than that of the 14% CP diet group.

**Table 6:** Effects of dietary crude protein (CP) levels on the carcass traits of Huanjiang mini-pigs from 5- to 10-kg^1^.

**Items**	**CP levels, %**					**SEM**	***p-*** **values^2^**		
	**14**	**16**	**18**	**20**	**22**		**D**	**L**	**Q**
Carcass straight length, cm	46.00	45.29	45.38	44.71	44.67	0.67	0.97	0.50	0.80
Carcass slanting length, cm	42.13	40.43	41.25	41.14	41.67	0.89	0.98	0.94	0.90
Eye muscle area, cm^2^	7.96	6.75	7.58	7.79	7.67	0.45	0.94	0.93	0.91
Backfat thickness, mm	11.73	10.96	10.25	10.51	11.85	0.38	0.62	0.82	0.28
Carcass weight, kg	7.95	7.17	7.01	7.02	7.57	0.24	0.66	0.50	0.29
Slaughter rate, %	59.35	57.00	56.11	55.30	54.38	0.87	0.45	0.06	0.15
Lean meat percentage, %	45.67^B^	43.14^B^	49.46^A^	46.21^AB^	46.00^AB^	0.59	<0.01	0.36	0.37
Fat percentage, %	19.16^AB^	22.31^A^	15.44^B^	17.64^B^	15.76^B^	0.77	0.02	0.04	0.13
Skin rate, %	14.71^a^	11.75^b^	11.29^b^	11.36^b^	12.31^ab^	0.45	0.06	0.07	0.01
Bone percentage, %	20.46^C^	22.81^BC^	23.81^AB^	24.79^A^	25.92^A^	0.51	<0.01	<0.001	<0.001

1 *Different capital letters indicate differed significantly among the treatments (p < 0.05), and different lowercase indicate a trend of difference (0.05 ≤ P < 0.10; n = 6–8*.

2 *D, diet; L, linear; Q, quadratic*.

As shown in Table 7, during the 10- to 20-kg growth stage, the slaughter rate was decreased (*p* < 0.05), whereas the skin rate was increased (*p* < 0.05) as the dietary CP level increased. Furthermore, the slaughter rate in the 12% CP diet group was higher (*p* < 0.05) and the skin rate in the 20% CP diet group was higher (*p* < 0.05) when compared with the other groups.

**Table 7:** Effects of dietary crude protein (CP) levels on the carcass traits of Huanjiang mini-pigs from 10- to 20-kg^1^.

**Items**	**CP levels, %**					**SEM**	***p-*** **values^2^**		
	**12**	**14**	**16**	**18**	**20**		**D**	**L**	**Q**
Carcass straight length, cm	62.00	59.00	62.57	61.43	61.57	0.44	0.21	0.62	0.76
Carcass slanting length, cm	61.38	59.75	59.71	59.57	59.43	0.46	0.65	0.20	0.33
Eye muscle area, cm^2^	15.51	13.10	14.55	15.01	14.21	0.35	0.24	0.88	0.76
Backfat thickness, mm	1.85	1.80	1.77	1.72	1.72	0.05	0.89	0.33	0.61
Carcass weight, kg	16.59	15.39	16.20	17.23	15.74	0.31	0.50	0.92	0.99
Slaughter rate, %	70.00^A^	64.92^B^	65.56^B^	66.12^B^	64.02^B^	0.55	<0.01	<0.01	<0.01
Lean meat percentage, %	43.25	44.67	42.18	42.79	42.78	0.42	0.40	0.33	0.63
Fat percentage, %	28.08	25.29	27.49	25.90	24.47	0.56	0.18	0.15	0.36
Skin rate, %	11.67^C^	12.73^B^	12.55^BC^	12.73^B^	14.34^A^	0.20	<0.01	<0.01	<0.01
Bone percentage, %	17.26	18.68	18.28	18.25	19.03	0.24	0.23	0.07	0.17

1 *Different capital letters indicate differed significantly among the treatments (p < 0.05); n = 7–9*.

2 *D, diet; L, linear; Q, quadratic*.

As shown in Table 8, during the 20- to 30-kg growth stage, as dietary CP levels increased, the eye muscle area in the 10% CP diet group was lower (*p* < 0.05) than those of the other groups. However, the slaughter rates of the 10 and 12% CP diet groups were higher (*p* < 0.05) than those of the 16 and 18% CP diet groups. In addition, the 18% CP diet group had a higher trend (*p* = 0.052) of the bone percentage than the 10, 12, and 14% CP diet groups and had a higher trend (*p* = 0.09) of the skin rate than the 12% CP diet group.

**Table 8:** Effects of dietary crude protein (CP) levels on the carcass traits of Huanjiang mini-pigs from 20- to 30-kg^1^.

**Items**	**CP levels, %**					**SEM**	***p-*** **values^2^**		
	**10**	**12**	**14**	**16**	**18**		**D**	**L**	**Q**
Carcass straight length, cm	65.71	66.88	66.57	68.13	66.63	0.47	0.62	0.38	0.45
Carcass slanting length, cm	63.14	64.75	63.57	65.13	64.38	0.50	0.74	0.46	0.67
Eye muscle area, cm^2^	13.55^C^	17.00^AB^	15.45^BC^	19.14^A^	17.99^AB^	0.57	0.01	<0.01	0.01
Backfat thickness, mm	2.06	2.24	2.14	2.03	2.00	0.06	0.68	0.38	0.47
Carcass weight, kg	19.77	20.21	18.92	20.06	18.82	0.34	0.60	0.39	0.65
Slaughter rate, %	63.35^A^	63.55^A^	61.22^AB^	59.51^B^	60.45^B^	0.47	0.01	<0.01	<0.01
Lean meat percentage, %	41.66	41.26	42.13	42.81	41.13	0.51	0.84	0.89	0.80
Fat percentage, %	27.85	29.38	27.71	26.13	25.40	0.60	0.22	0.045	0.09
Skin rate, %	13.35^a^	11.73^b^	12.66^ab^	13.11^ab^	13.67^a^	0.25	0.09	0.20	0.07
Bone percentage, %	17.14^b^	17.64^b^	17.51^b^	17.95^ab^	19.80^a^	0.32	0.052	<0.01	0.01

1 *Different capital letters indicate differ significantly among the treatments (p < 0.05), and different lowercase indicate a trend of difference (0.05 ≤ P < 0.10; n = 7–8*.

2 *D, diet; L, linear; Q, quadratic*.

### Relations Between Dietary CP Levels and Carcass Traits

**Figure 3:**
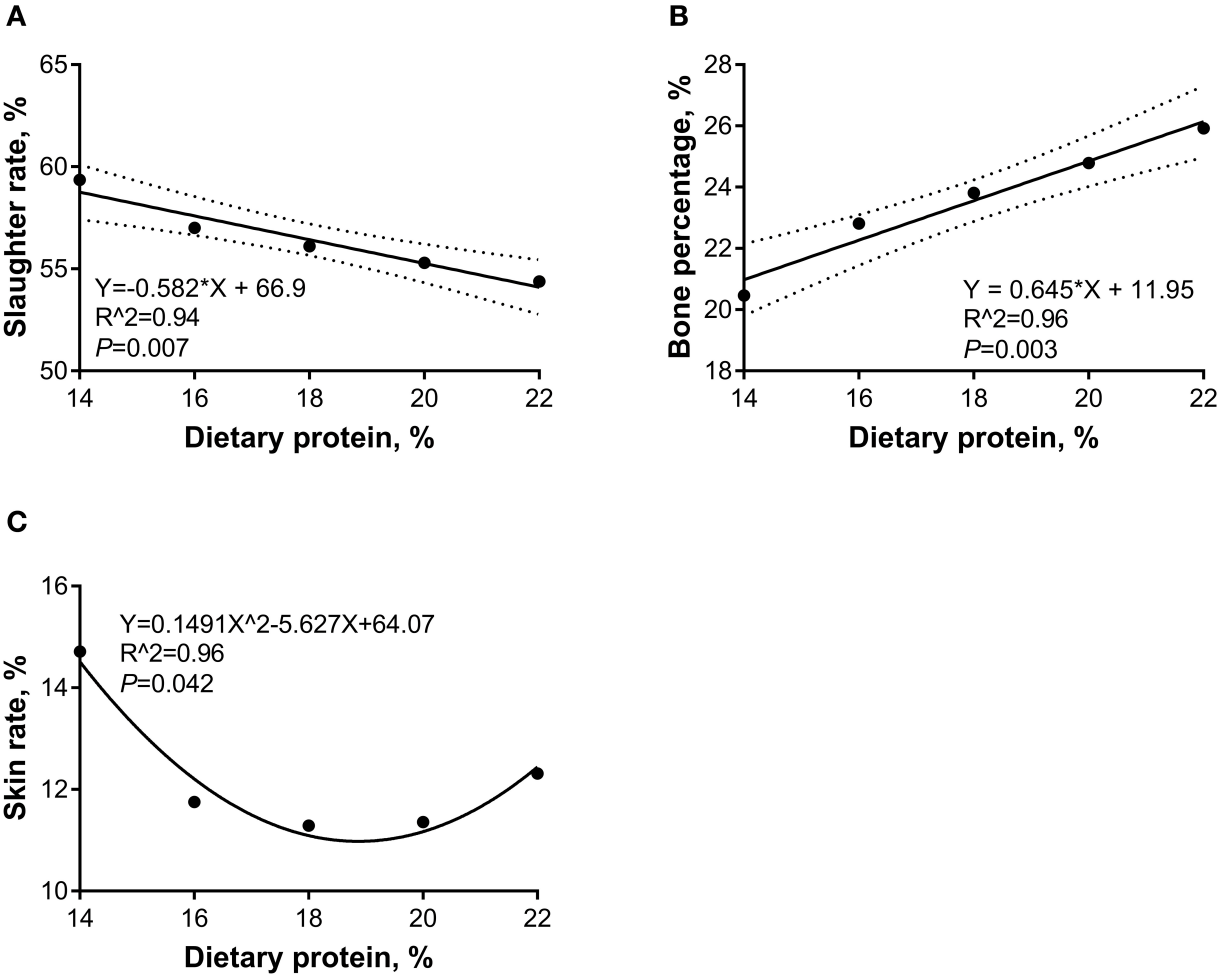
Regression equation for the relations between dietary crude protein (CP) levels and slaughter rate **(A)**, bone percentage **(B)**, and skin rate **(C)** of 5- to 10-kg Huanjiang mini-pigs. The dotted lines in **(A,B)** represent the confidence interval of 95%, and the minimal point for **(C)** is (18.87, 10.98).

**Figure 4:**
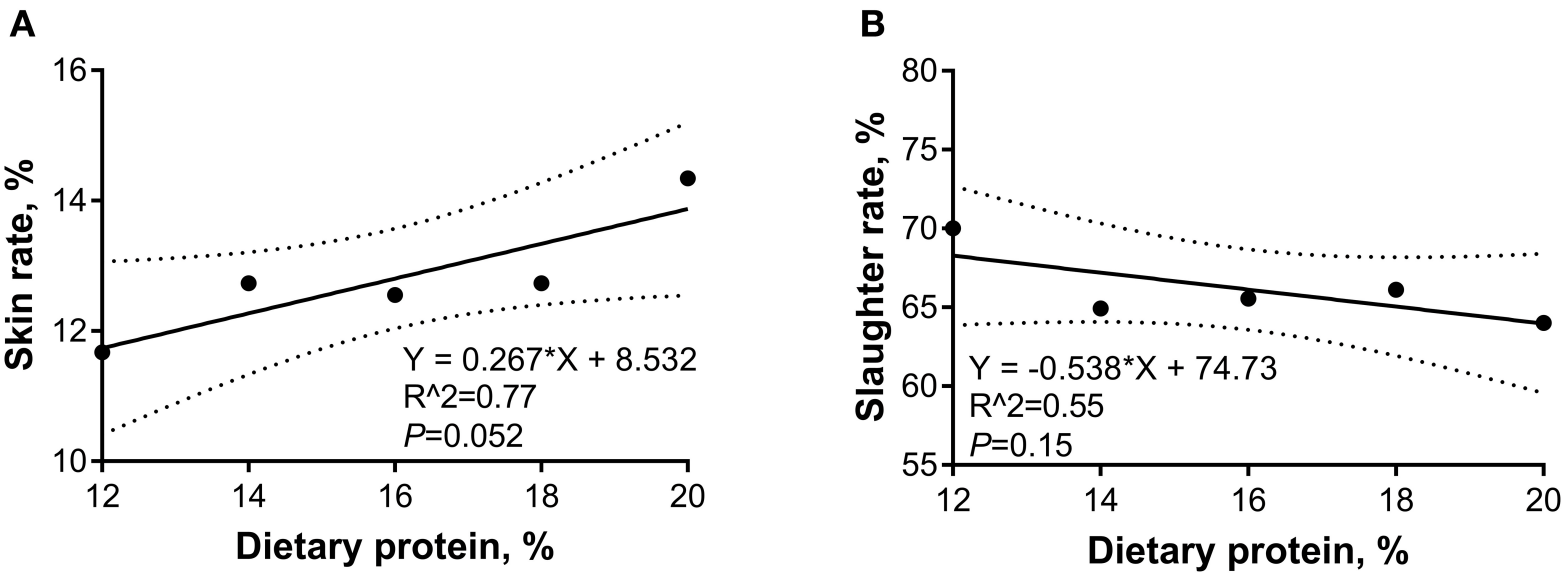
Regression equation for the relations between dietary crude protein (CP) levels and slaughter rate **(A)** and skin rate **(B)** of 10- to 20-kg Huanjiang mini-pigs. The dotted lines in **(A,B)** represent the confidence interval of 95%.

**Figure 5:**
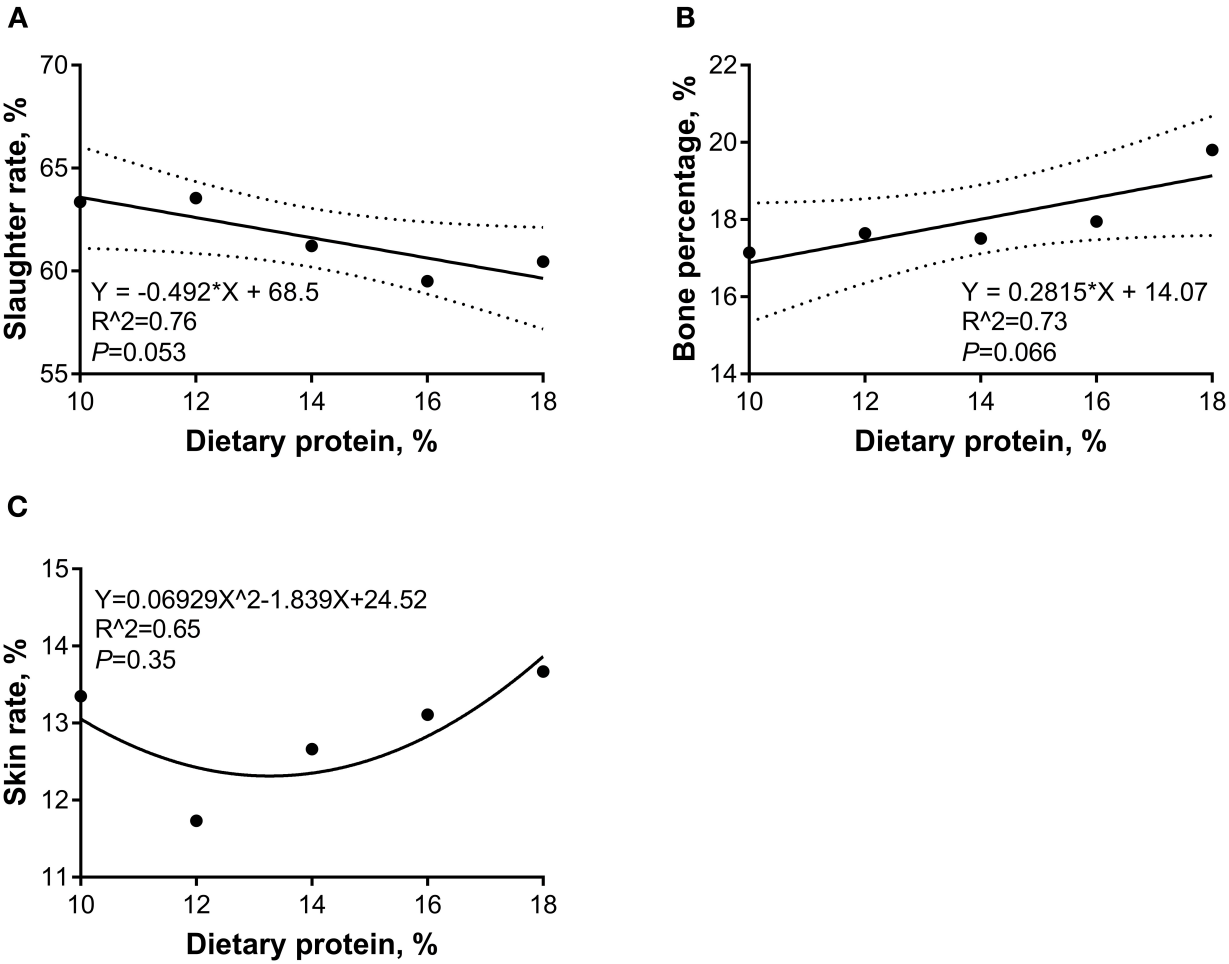
Regression equation for the relations between dietary crude protein (CP) levels and slaughter rate **(A)**, bone percentage **(B)**, and skin rate **(C)** of 20- to 30-kg Huanjiang mini-pigs. The dotted lines in **(A,B)** represent the confidence interval of 95%, and the minimal point for **(C)** is (13.27, 12.32).

The linear and quadratic regression analyses were conducted to investigate the relations between the dietary CP levels and carcass traits. As shown in Figure 3, during the 5- to 10-kg growth stage, as the dietary CP level increased, the slaughter rate was decreased (*p* < 0.05) linearly, and the bone percentage increased (*p* < 0.05) linearly, whereas the skin rate showed a quadratic relation and the minimal points were 18.87 and 10.98%, respectively. As shown in Figure 4, during the 10- to 20-kg growth stage, as the dietary CP level increased, the slaughter rate was decreased, and the skin rate was increased (*p* < 0.05) linearly. As shown in Figure 5, during the 20- to 30-kg growth stage, as the dietary CP level increased, the slaughter rate was decreased and the bone percentage was increased linearly (*p* < 0.05), while the skin rate showed a quadratic relation and the minimal point was (13.27 and 12.32%).

### Correlations Between Carcass Traits

**Figure 6:**
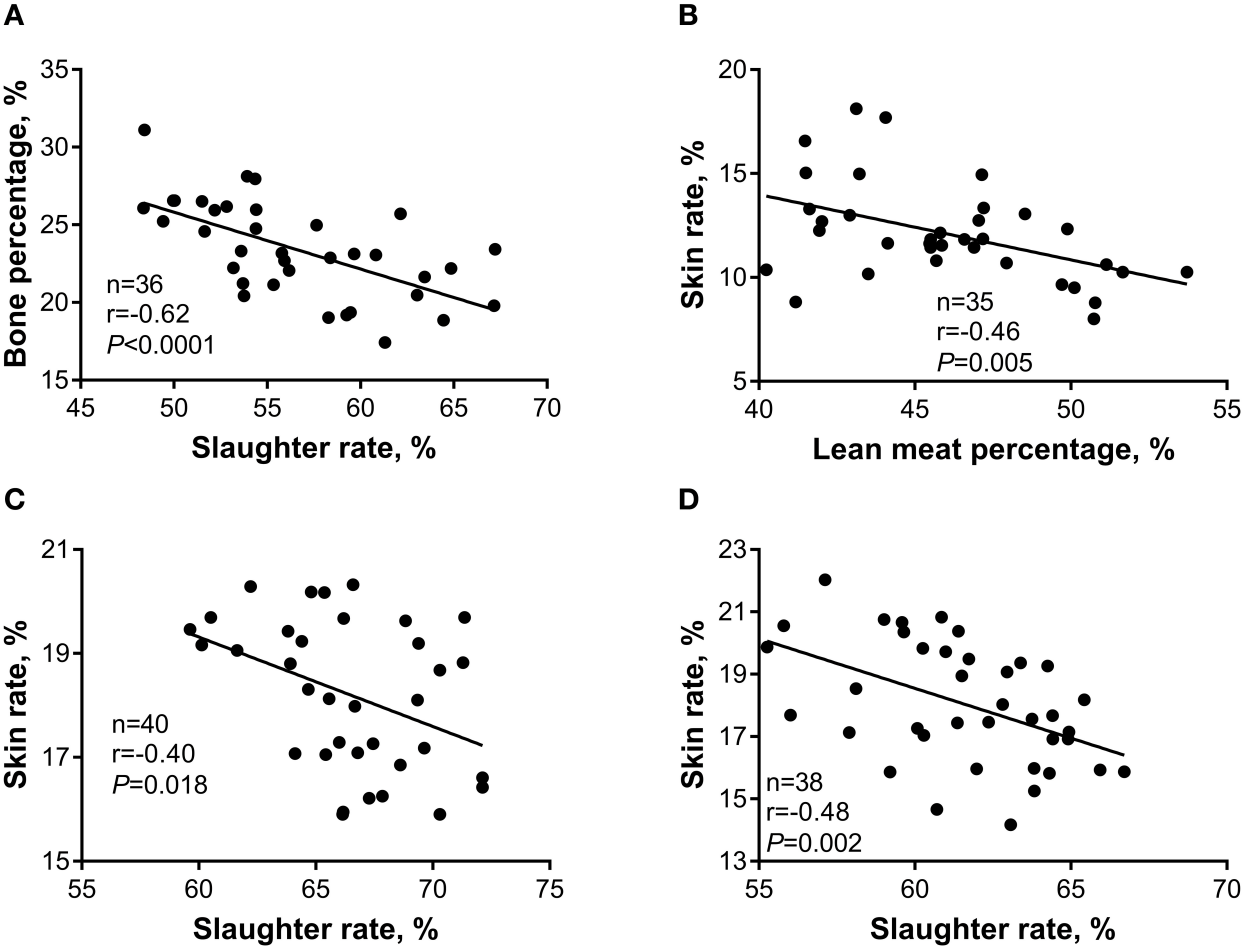
Pearson correlation analysis for the relations between bone percentage and slaughter rate (**A**, 5- to 10-kg) and between skin rate and lean meat percentage (**B**, 5- to 10-kg), and between skin rate and slaughter rate (**C**, 10- to 20-kg; **D**, 20- to 30-kg) of Huanjiang mini-pigs.

The correlations between the slaughter rate and skin rate and bone percentage, as well as the correlation between the skin rate and lean meat percentage, were further investigated to explore their potential associations. As shown in Figure 6, during the 5- to 10-kg growth stage, a strong negative correlation (*r* = −0.62, *p* < 0.001) was observed between the slaughter rate and bone percentage (Figure 6A), as well as a strong negative correlation (*r* = −0.46, *p* = 0.005) between the skin rate and lean meat percentage (Figure 6B). During the 10- to 20-kg growth stage, a strong negative correlation (*r* = −0.40, *p* = 0.018) was observed between the skin rate and slaughter rate (Figure 6C). Similarly, a strong negative correlation (*r* = −0.48, *p* = 0.002) was observed between the skin rate and slaughter rate during the 20- to 30-kg growth stage (Figure 6D).

## Discussion

It is well-known that the nutrient requirements for different pig breeds are not the same and are influenced by genotype, physiological status, environmental conditions, and other factors. Generally, the nutrient requirements of pigs are evaluated using a mathematical modeling method in terms of their specific performance parameters, such as growth performance and protein accretion rate (22). Therefore, the present study investigated the optimal dietary CP level for Huanjiang mini-pigs by regression equations established based on growth performance, serum biochemical parameters, and carcass traits. Our data showed that the optimal dietary CP levels for the 5- to 10-kg, 10- to 20-kg, and 20- to-30 kg growth stages were 18.42, 16.70, and 14.00%, respectively. These findings suggested that the dietary CP levels derived from the Chinese National Feeding Standard for Swine (19) or the National Research Council National Research Council (18) were unsuitable for Huanjiang mini-pigs during the early growth period, of which the values recommended were too high to achieve their optimal growth potential.

In the present study, a quadratic response to the dietary CP levels was observed for the ADFI, ADG, and F/G of Huanjiang mini-pigs during different growth stages, and the changes of ADG and F/G were in agreement with the reports of Tyler et al. (23) and Hansen and Lewis (24). Considering the same diet type (corn–soybean meal) used in these studies, it could be speculated that the response of various pig breeds to the dietary CP levels might have mainly depended on a diet type, which warrants further study. Recently, a similar study also indicated that increasing the dietary CP levels (16.9–20.9%) resulted in a linear decrease in ADG and a linear increase in BUN concentration (25), which were consistent with our present results. The BUN concentration is an indicator of protein utilization and is mostly dependent on the balance of amino acids (AAs) (26, 27). Besides, NH_3_-N is a substance that originated from the intestinal microbiota-fermented protein and AAs, and its serum concentration could also reflect the utilization of dietary protein. A lower serum NH_3_-N concentration was found in pigs fed with a lower CP diet (28, 29). Therefore, the increased serum UN or NH_3_-N concentration combined with the impaired growth performance in the present study suggested an excessive dietary CP level. The serum TP and albumin contents reflect the dietary CP level and the utilization of protein by the animal body, and an increased serum TP content was observed when pigs' protein utilization increased (29, 30). In the present study, as the dietary CP levels increased, a decreased serum TP was observed during the 5- to 10-kg growth stage (14–22% CP), while an increased serum albumin content was observed during the 20- to 30-kg growth stage (10–18% CP). These findings were contrary to those reported by Zhao et al. (29), and the different dietary CP levels could be the reason for this difference.

In the present study, although the curve's change of ADG was in line with the trend of ADFI of pigs, the response of ADFI of commercial pigs to the dietary CP levels depended on sex (24). Whether the ADFI of Chinese indigenous pigs with mixed sexes has the same response to the identical dietary CP levels should be determined in the future. The ADFI of pigs is influenced by lots of factors, the energy density of which is the most important (31, 32). Our previous studies showed that the ADFI and ADG of Huanjiang mini-pigs were 860 and 285 g during the 10- to 25-kg growth stage, respectively (33), which were lower than those in the present study. The difference may be attributed to the difference in the dietary energy density. The dietary energy density in the present study was higher by 5% than that of the previous study (14.18 MJ/kg DE vs. 13.48 MJ/kg DE), which could be a reason that influenced the growth performance of pigs (34). Dietary CP level is another important factor to regulate the ADFI of pigs, which referred to dietary AA imbalance (32). Dietary CP level was 16.13% for pigs during the 10- to 25-kg growth stage in a previous study (33), which was close to the level (16.70%) predicted by the mathematical modeling method in the present study. It would increase the feed intake of growing pigs when feeding a diet deficiency in Lys or Thr (35). However, dietary Lys and Thr levels and feed intake of the present study were higher than those reported by Cai et al. (33). The Lys and Thr are the primary limiting AAs for pigs, and the feed intake would be suppressed by a severe deficiency or excessive in dietary limiting AAs (36). Taken together, we speculated that the Lys in diet of the previous study might be severely deficient as a lower feed intake was observed during the 10- to 25-kg growth stage. Certainly, it was also suggested that the application of the ideal AA pattern in the diet of Huanjiang mini-pigs would be beneficial for developing their growth potential. Dietary Lys deficiency or excessive would decrease the nutrient digestibility (37), and the F/G in our previous study was higher by 36.5% than that of the present result (4.04 vs. 2.96) (33). Furthermore, increasing the dietary CP levels linearly decreases the nutrient digestibility of pigs (38). Therefore, the appearing difference could be largely ascribed to a lower nutrient digestibility of pigs in the previous study. Whether or not the other limiting AAs have a promotion effect on the nutrient digestibility of Huanjiang mini-pigs could be a significant research interest in the future. Decreased feed efficiency or nutrient digestibility could be largely due to a higher incidence of diarrhea, which could be indirectly reflected by the fecal nitrogen content (39). In the present study, excessive dietary CP significantly increased the fecal nitrogen content and the diarrhea rate (unpublished data). Therefore, the impaired growth performance of pigs, such as the reduced ADG, could be ascribed to a higher incidence of diarrhea.

In the present study, dietary CP levels had a distinct effect on the carcass traits. As dietary CP levels increased, the slaughter rate of pigs during different growth stages decreased linearly, which was in agreement with the previous studies (24, 40). Conversely, several previous studies showed that reducing the CP level by 4% based on a normal CP diet and balanced with crystalline AAs had no significant effects on the slaughter rate (41, 42). The different breed and growth phases of pigs might be the possible reason for the different results that appeared among these studies. A quadratic response to dietary CP levels was observed in the skin rate of pigs, and the predicted dietary CP levels (18.87 and 13.27% for pigs during the 5- to 10-kg and the 20- to 30-kg growth stages, respectively) were close to that for optimal growth performance of pigs in the present study, suggesting that the response of skin rate to dietary CP levels was consistent with that of the growth performance of Huanjiang mini-pigs. During the 20- to 30-kg growth stage (10–18% CP), the eye muscle area was increased with increasing dietary CP levels. On the contrary, previous studies have shown that dietary CP levels had no effect on the eye muscle area in cattle (11–14% CP) or commercial pigs (15–17% CP) (43, 44). The species or breed difference might be the possible reason for the inconsistent results, but it warranted further research to determine. During the 5- to 10-kg growth period, increasing the dietary CP levels decreased the fat percentage. Similarly, the fat deposition of calves decreased as dietary CP levels increased irrespective of growth stage (45), and reduced fat deposition in pigs fed with high dietary protein was mainly by depression of lipogenic gene expression (46).

In the present study, no significant difference was observed in carcass weight, carcass length, and backfat thickness of pigs during different growth stages, which is in accordance with a previous study (42). However, a strongly negative correlation (*p* < 0.05) was observed between the slaughter rate and bone percentage (*r*_bone percentage_ = −0.62, 5–10 kg) and skin rate (*r*_skin rate_ = −0.40, 10–20 kg; *r*_skin rate_ = −0.48, 20–30 kg). As live weight increases, there are significant increases in slaughter rate (47), which is in accordance with that reported by Daza et al. (48). These findings suggested that the increase in the slaughter rate of Huanjiang mini-pigs might cause the sacrificed development of skin or skeleton during the early growth stage.

## Conclusions

In summary, as dietary CP levels increased, the F/G quadratically decreased during the 5- to 10-kg growth stage, the ADG quadratically increased during the 10- to 20-kg growth stage, and the ADFI and ADG quadratically increased during the 20- to 30-kg growth stage. Increasing the dietary CP levels linearly decreased the slaughter rate but linearly increased the bone percentage of Huanjiang mini-pigs during different growth stages. Taken together, it is the first time to provide the optimal dietary CP levels 18.42, 16.70, and 14.00% for Huanjiang mini-pigs during the 5- to 10-kg, 10- to 20-kg, and 20- to 30-kg growth stages, respectively.

## Data Availability Statement

The data used to support the findings are included in the article/Supplementary Material, further inquiries can be directed to the corresponding author/s.

## Ethics Statement

The animal study was conducted and according to the Chinese guidelines for animal welfare and experimental protocols and approved by the Animal Care and Use Committee of Institute of Subtropical Agriculture, Chinese Academy of Sciences (No. ISA-2019-4-29).

## Author Contributions

XZ, XK, and YY designed the experiment and contributed to the interpretation of the data. XZ was responsible for the original draft written, modification of the manuscript, feeding of the animal, sample collection, and lab analysis. HD participated in sample collection for the trials. YL was involved in feeding of the animal during the trial 1. PH provided the kind assistance during lab analysis. JD provided constructive suggestions on the animal feeding. All authors contributed to the article and approved the submitted version.

## Funding

This present study was jointly supported by the Production and Research Talent Support Project of the CAS Wang Kuancheng Initiative Talent Program, Special Funds for Construction of Innovative Provinces in Hunan Province (2019RS3022), the STS regional key project of the Chinese Academy of Sciences (KFJ-STS-QYZD-052), and the Tianjin Synthetic Biotechnology Innovation Capacity Improvement Project (TSBICIP-CXRC-038).

## Conflict of Interest

The authors declare that the research was conducted in the absence of any commercial or financial relationships that could be construed as a potential conflict of interest.

## Publisher's Note

All claims expressed in this article are solely those of the authors and do not necessarily represent those of their affiliated organizations, or those of the publisher, the editors and the reviewers. Any product that may be evaluated in this article, or claim that may be made by its manufacturer, is not guaranteed or endorsed by the publisher.

## Abbreviations

AA: amino acid
ADFI: average daily feed intake
ADG: average daily gain
BW: body weight
CP: crude protein
DE: digestible energy
F/G: feed to gain ratio
Lys: lysine
ME: metabolic energy
Met: methionine
NRC: National Research Council
SID: standard ileal digestible
Thr: threonine
TP: total phosphorus
Trp: tryptophan
UN: urea nitrogen.
