# Supplementary material for: Effects of Different Dietary Protein Levels on the Growth Performance, Serum Biochemical Parameters, Fecal Nitrogen, and Carcass Traits of Huanjiang Mini-Pigs
Source: Front Vet Sci. 2021 Dec 20;8:777671. doi: 10.3389/fvets.2021.777671 (PMC8720777; doi:10.3389/fvets.2021.777671)
Supplement: Supplementary file 1 [file Data_Sheet_1.docx]

Supplemental material


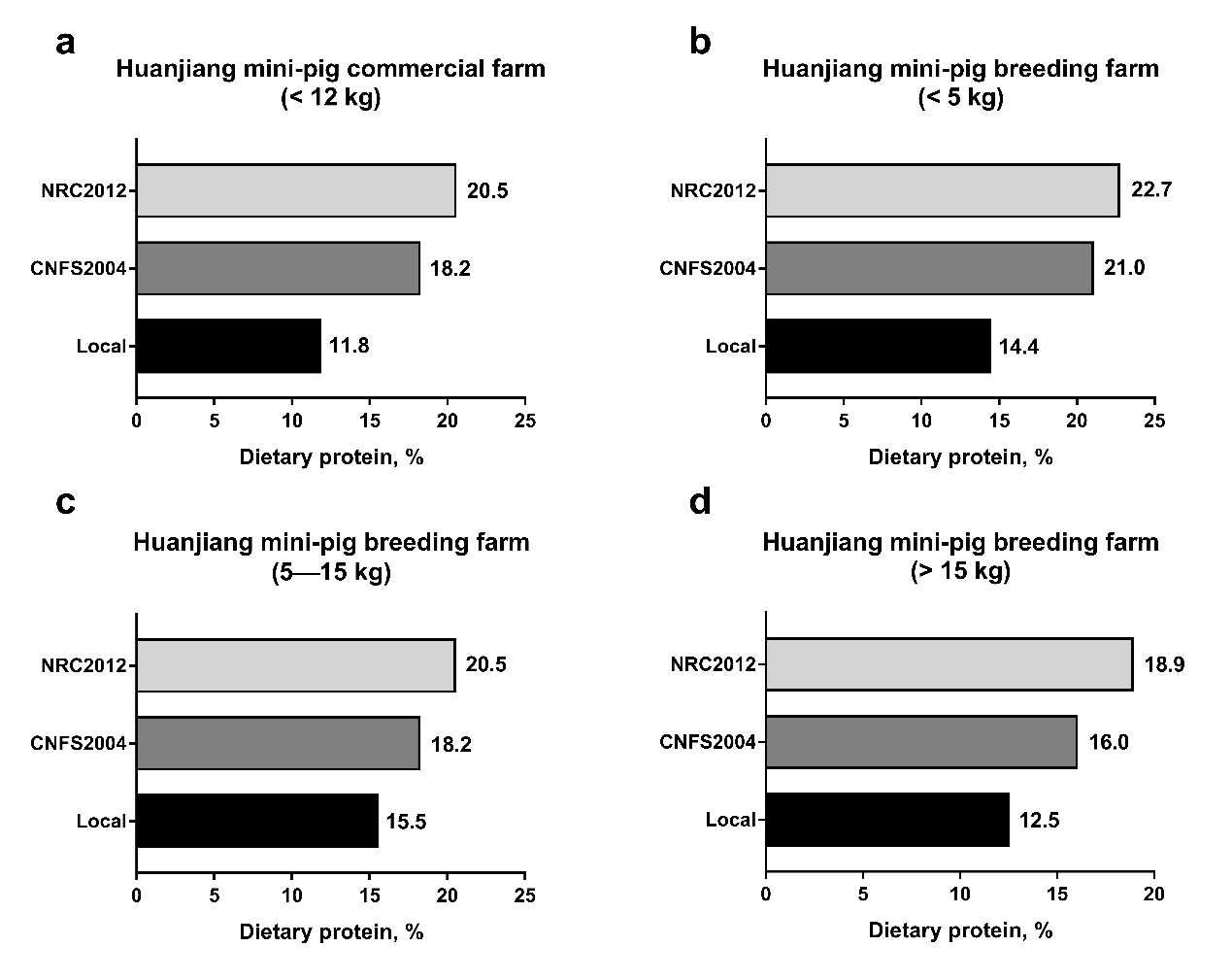


**Fig. S1. Comparison of dietary crude protein CP levels in formulas used by local pig farms of Huanjiang mini-pig and those recommended by the National Research Council(NRC,2012) or Chinese national feeding standard for swine (CNFS, 2004).**

(a) indicates the formula derived from a commercial farm and is appropriate for pigs less than 12 kg; (b), (c), and (d) indicate the formula derived from the breeding farm and is appropriate for weaned pig < 5 kg, 5 to 15 kg, and > 15 kg growing pig, respectively. The dietary CP levels of local pig farms were calculated according to the formulas for Huanjiang mini-pigs in different growth period and the nutrient levels of ingredients in reference to values of NRC (2012).
